# Evaluation of a guided continuous quality improvement program in community pharmacies

**DOI:** 10.1186/s40545-017-0114-x

**Published:** 2017-09-05

**Authors:** Chanadda Chinthammit, Michael T. Rupp, Edward P. Armstrong, Tara Modisett, Rebecca P. Snead, Terri L. Warholak

**Affiliations:** 1 Department of Pharmaceutical Sciences, College of Pharmacy, University of Arizona, Tucson, AZ USA; 2 Department of Pharmacy Practice, College of Pharmacy, Midwestern University, Glendale, AZ USA; 3 Department of Pharmacy Practice and Science, College of Pharmacy, University of Arizona, Tucson, AZ USA; 4 Alliance for Patient Medication Safety, Richmond, VA USA

**Keywords:** Patient safety, Quality improvement, Incident reporting and analysis, Safety culture, Rasch model, Pharmacies

## Abstract

**Background:**

The importance of creating and sustaining a strong culture of patient safety has been recognized as a critical component of safe medication use. This study aims to assess changes in attitudes toward patient safety culture and frequency of quality-related event (QRE) reporting after guided implementation of a continuous quality improvement (CQI) program in a panel of community pharmacies in the United States (U.S.).

**Methods:**

Twenty-one community pharmacies volunteered to participate in the project and were randomly assigned to intervention or control groups. Pharmacy staff in the intervention group received guided training to ensure full implementation of a CQI program while those in the control group partially implemented the program. Pharmacy staff in both groups completed retrospective pre-post safety culture questionnaires and reported medication errors and near misses that occurred in their practices. Rasch analysis was applied to assess questionnaire validity and reliability and to confirm if the ordinal level data approximated interval level measures. Paired t-tests and repeated measure analysis of covariance tests were subsequently used to compare observed changes in the attitudes of subjects and frequency of QREs reporting in intervention and control groups.

**Results:**

Sixty-nine employees completed the questionnaire, a 43.9% response rate. Improvement in attitudes toward patient safety was statistically significant in the intervention group in six domains: staff, training, and skill (*p* = 0.017); patient counseling (*p* = 0.043); communication about mistakes (*p* < 0.001); response to mistakes (*p* < 0.001); organizational learning – continuous improvement (*p* < 0.001); and overall patient safety perceptions (*p* = 0.033). No significant differences were observed in QRE reporting rates between intervention and control groups. However, differences were observed in the types of QREs reported (e.g., incorrect safety cap) and the point in the prescription processing workflow where a QRE was detected (e.g., partner check station, and drug utilization review station) in the intervention group (*p* < 0.001).

**Conclusion:**

Guided CQI program implementation increased the self-reported patient safety culture attitudes among staff.

**Electronic supplementary material:**

The online version of this article (10.1186/s40545-017-0114-x) contains supplementary material, which is available to authorized users.

## Background

In its 1999 report, *To Err is Human: Building a Safer Health System,* the U.S. Institute of Medicine (IOM) highlighted the need for a cultural change in how health care organizations and providers approach medical and medication errors [1]. This call heightened error awareness and made patient safety an issue of concern for all healthcare providers and institutions. In the years since the IOM report was issued, many quality improvement programs have been implemented to reduce the incidence of medication errors [1–7]. A study conducted in Canadian community pharmacies found that implementation of continuous quality improvement (CQI) resulted in improvements in 7 key areas that are known to be systems-based sources of medication errors including: patient information; drug information; communication of drug orders; drug labeling and packaging; and drug standardization and distribution [7].

Beyond such targeted strategies, the importance of creating and sustaining a strong culture of patient safety has been recognized as a critical component of safe medication use [8, 9]. A culture of patient safety refers to the beliefs, values and norms regarding how members of an organization should behave and includes policies, procedures and processes to improve quality and safety [10–12]. CQI process use is central to creating a culture of patient safety within healthcare organizations [13]. Thus, the CQI philosophy must be embraced throughout the organization and a systematic CQI program must be implemented to analyze the causes of medication errors and to create strategies to prevent future errors [13]. However, this cannot happen unless the organization has a successful, sustainable safety culture.

Pharmacy Quality Commitment (PQC™) is a standardized, 5-step CQI program that was developed to assist pharmacy staff to document, monitor and analyze quality-related events (QREs), determine their likely cause(s) and implement changes to prevent future QREs [14]. The term QRE includes both errors (i.e., mistakes that reach the patient) and near misses that occur in the pharmacy (i.e., mistakes caught before they reach the patient).

During the past decade in the U.S., some states have adopted laws requiring pharmacies to implement quality assurance programs [15]. Given this trend, it is reasonable to ask how implementation of quality assurance programs like PQC™ affects the achievement of desired objectives. Specifically, the objective of this study was to assess the effect of full, guided implementation of the CQI program on: 1) safety culture attitudes; 2) incidence of reported medication QREs when compared to partial self-implementation by pharmacy staff.

## Methods

### Design and participants

**Fig. 1:**
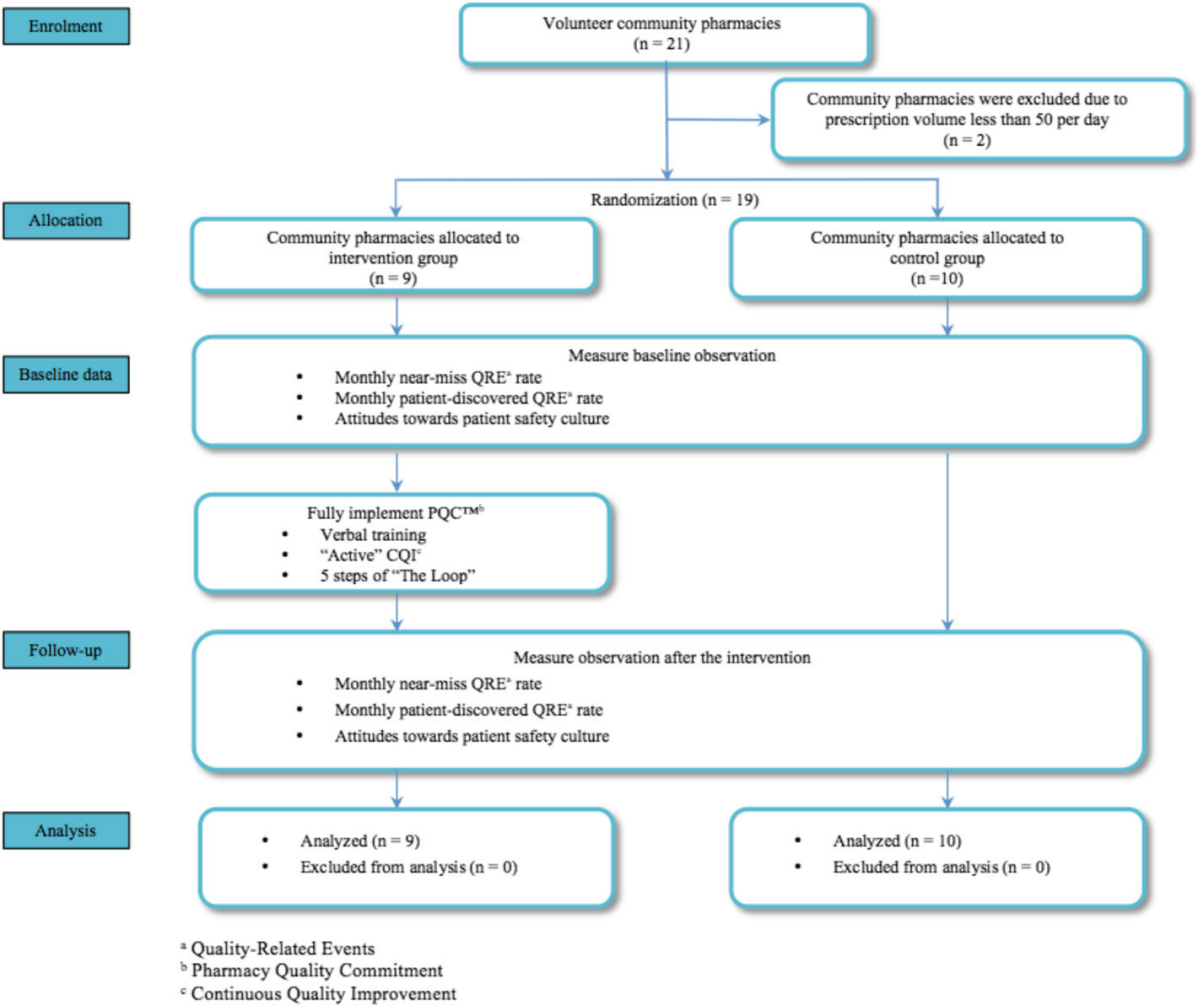
Flow of participants through the study (*N* = 21)

This randomized, controlled, parallel-group study was conducted in 21 volunteer community pharmacies in the United States. The pharmacies volunteered because their staff wished to learn how to more fully implement their CQI program. The pharmacies in the sample were randomized into intervention and control groups (Fig. 1). Intervention pharmacies were provided a training session to facilitate full program implementation (Steps 1–5 as described below) while control pharmacies continued their standard practice, which included only Steps 1 and 2.

- Step 1. “Establish and Communicate Work Flow” - pharmacies adopt best practices and standardized workflow guidelines.
- Step 2. “Collect QREs” - pharmacies implement use of standardized forms and instructions for QRE tracking and are able to access a private, secure, online system for quick documentation.
- Step 3. “Analyze QREs and Process Breakdowns” - data entered into the online reporting portal in Step 2 are analyzed. The portal helps the user convert the pharmacy’s data to CQI intelligence that can be interpreted by safety managers and easily presented to the pharmacy’s staff by assisting with performance of detailed analytics and production of illustrative charts/graphs.
- Step 4. “Formulate Plan for Improvement” - CQI intelligence gleaned from previous steps are used as the basis for developing actionable improvement plans.
- Step 5. “Implement New Process with Training”- the plans from the previous step are implemented and benchmarks are established to measure success.

Prior to the study, all pharmacies in this study routinely utilized Steps 1 and 2 of the CQI cycle but had not formally operationalized Steps 3–5. “Standard practice” was therefore defined as reporting QREs daily but not completing the other portions of the CQI cycle.

### Components of the intervention (i.e., training session)

The pharmacy managers and staff who were responsible for risk management in the intervention pharmacies attended a training session presented by the study’s primary investigator who was well trained in quality improvement and risk management area. In addition, two faculty members who were specialized in the same area and a practice community pharmacist reviewed the training materials and course. Over a period of 2 weeks, the designated risk manager at each pharmacy trained their pharmacists and staff to fully implement the 5-step PQC™ system. The training session was developed using the Dick and Carey instructional design model, a systematic approach that embraces interaction among instructors, learners, materials, learning activities, delivery system, and learning and performance environments and brings them to work together to achieve learning outcomes [16]. All training materials were reviewed by educational and CQI experts before delivery and based on their recommendations revisions were made before training subjects.

The intervention involved training the pharmacy staff to operationalize all 5 steps on the PQC™ cycle workflow [14] during four face-to-face sessions (see session I to IV as described below).

- Session I. Overview of PQC: how to use the PQC™ program to analyze the error data and interpret the results from graph and bar charts as well as how to use daily PQC™ process related event report form to facilitate root cause analysis during the peer review process to diagnose the possible causes of QREs.
- Session II. Analysis of QREs and procedures: Step 3 and error reduction strategies to handle different types of safety issues (e.g., standardization, double checks, reminder & checklists).
- Session III. How to formulate plan for improvement: Step 4 and how to train pharmacists and pharmacy staff effectively and monitor the new process.
- Session IV. New processes implementation with training: Step 5. This session also included a mock peer review meeting during which participants role played leading their team in conducting a root cause analysis.

### Data collection

Data were collected between July and August 2014. The study measured two types of QREs; those detected by the pharmacy staff before the medication reached the patient (i.e., “near misses”) and those discovered after the patient left the pharmacy with the medication (i.e., “error”). All QRE data were reported via the PQC™ reporting portal to Alliance for Patient Medication Safety (APMS) which is a Patient Safety Organization (PSO). The Daily PQC™ process related events report form was used for data collection. Data were transferred to the researchers in a de-identified format. No pharmacy-specific identifiers and no specific patient or provider identifiers were used in reporting.

A week prior to study initiation, the primary investigator visited each pharmacy to explain the study expectations and request informed consent from participants. At this time the paper-based safety culture attitudinal questionnaires were distributed which were subsequently collected at the end of the study. The Agency for Healthcare Research and Quality’s (AHRQ) Pharmacy Survey on Patient Safety Culture was modified into a retrospective pre-post format and used to assess changes in pharmacy patient safety culture [17]. Pharmacists and pharmacy technicians in both intervention and control pharmacies were asked to complete the questionnaire. The questionnaire was divided into 5 sections covering 11 domains including: physical space and environment; teamwork, staff training and skills; communication openness; patient counseling; staffing, work pressure and pace; communication about prescription across shifts; response to mistakes; organizational learning – quality improvement; and overall perception of patient safety [17]. The university-based Institutional Review Board approved the study protocol.

### Data analysis

The first main outcome measure of interest in this project was the change in self-reported pharmacy staff attitudes toward patient safety culture. Rasch analysis was used to evaluate questionnaire, scale and item performance [18] and reliability was also assessed using Cronbach’s alpha. The Wolf and Chui method for pre-post analysis was used to account for construct shift bias where pre to post attitudinal responses were converted via Rasch analysis into interval level data (scores) for each person where the data fit the model [18]. These scores were used as the dependent variable in paired t-tests and repeated measure analysis of covariance (ANOVA) for each domain [19, 20] to compare the difference between the two groups. The standardized difference eq. (Z scores) was used to identify gaps in item difficulties (content gaps). Winsteps software (3.80.1: Linacre JM, Beaverton, OR, U.S.A.) was used for Rasch analyses.

The second outcome measure of interest was the change in QRE reporting rates (i.e., number of QREs per dispensed prescriptions) between pharmacies in the intervention and control groups. The outcome was compared using ANOVA. SAS statistical packages were utilized for data management and analyses. For all tests, an alpha of 0.05 was selected a priori.

## Results

### Attitude toward patient safety culture

Out of 157 staff working in the pharmacies, 112 (71.3%) responded to the retrospective pre-post Patient Safety Culture questionnaire. However, 69 (43.9%) respondents completed both pre- and post-intervention sections of the questionnaire. Respondent demographics are presented in Table 1. The preliminary questionnaire assessment indicated that the “negative” and “neutral” scale options were rarely utilized. Therefore, in order to optimize scale functionality, the categories of “disagree” and “strongly agree” and “rarely” and “never” were collapsed. The scale functioning for most domains met the requirement of category utilization after collapsing.

**Table 1:** Baseline characteristics of respondents

Characteristics	Intervention (*N* = 29)	Control (*N* = 40)	
	*N* (%)	*N* (%)	*p* ^†^
Pharmacy staff			1.000
Pharmacists	16 (55.2)	17 (42.5)	
Pharmacy staff	12 (41.4)	23 (56.7)	
Unknown	1 (3.4)	0 (0)	
Years worked in Pharmacy Setting			0.644
< 6 months	0 (0)	2 (5.0)	
6 months – 1 year	2 (6.9)	5 (12.5)	
1–3 years	6 (20.7)	10 (25.0)	
3–6 years	11 (37.9)	12 (30.0)	
6–12 years	6 (20.7)	5 (12.5)	
> 12 years	2 (6.9)	6 (15.0)	
Unknown	2 (6.9)	0 (0)	
Hours worked per week in Pharmacy Setting			0.472
1–16 h per week	0 (0)	3 (7.5)	
17–31 h per week	1 (3.5)	1 (2.5)	
32–40 h per week	23 (79.3)	28 (70.0)	
> 40 h per week	4 (13.8)	8 (20)	
Unknown	1 (3.5)	0 (0)	

^†^ *p* values were computed using Fisher’s Exact tests

Most of the items from the 11 domains fit the Rasch model requirements in both pre- and post-intervention periods. However, content gaps were identified between items (standardized scores [i.e., Z-scores] outside 2 and −2) in either pre- or post-intervention period. In Domain3 “Staff Training and Skills,” for instance, a content gap was identified between item A8 “Staff who are new to this pharmacy receive adequate orientation” (mean = 0.89, standard error = 0.22) and item A10 “Staff get enough training from this pharmacy” (mean = −0.11, standard error = 0.26) with a Z-score of 2.93. Additional information is available as an Additional file 1. Cronbach’s alpha for domains ranged from 0.76 to 1.00.

**Fig. 2:**
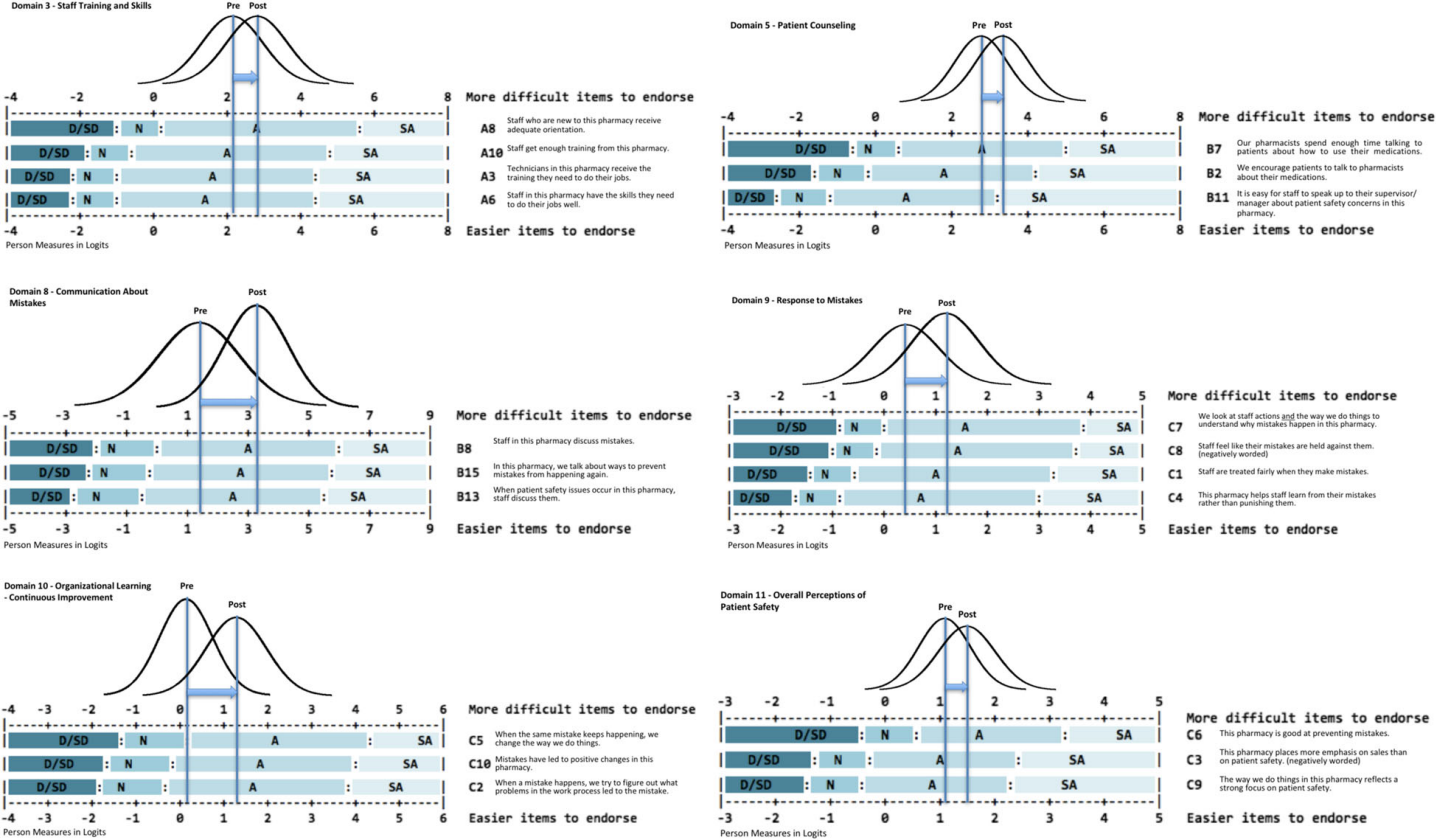
Rasch keyform map for patient safety attitude change for domains in which a significant change was seen pre to post intervention. Note: Pre: Pre-intervention period; Post: Post-intervention period; SD: Strongly disagree; D: disagree; N: Neither disagree or agree; A: Agree; SA: Strongly agree

Statistically significant pre-post improvements in attitude toward patient safety culture were found among intervention pharmacies for Domain 3 “Staff, Training, and Skill” (*p =* 0.017), Domain 5 “Patient Counseling” (*p* = 0.043), Domain 8 “Communication about Mistake” (*p* < 0.001), Domain 9 “Response to Mistakes” (*p* < 0.001), Domain 10 “Organizational Leaning-Continuous Improvement” (*p* < 0.001), and Domain 11 “Overall Perception of Patient Safety” (*p* = 0.033) (Fig. 2). In contrast, none of the domain scores changed significantly in the control group. Additional information is presented in Table 2.

**Table 2:** Change in attitude toward patient safety culture score and difference in score in intervention and control groups

		Intervention (*N* = 29)							Control (*N* = 40)							Difference in score changed rates		
		Pre		Post		Scores changed (Logit)			Pre		Post		Scores changed (Logit)					
	Domain	mean	S.D.	mean	S.D	mean	S.D.	*p* ^†^	mean	S.D.	mean	S.D	mean	S.D.	*p* ^†^	Beta	S.E.	*p* ^‡^
1	Physical Space and Environment	0.87	1.77	1.20	1.76	0.33	0.92	0.065	2.16	2.37	2.41	2.45	0.25	0.84	0.071	0.08	0.21	0.695
2	Teamwork	1.54	2.40	2.02	2.46	0.48	1.26	0.050	3.68	2.86	3.90	2.80	0.22	0.85	0.116	0.26	0.25	0.303
3	Staff Training and Skills	2.16	2.52	2.72	2.50	0.57	1.20	0.017	3.96	3.15	4.17	3.17	0.21	0.92	0.147	0.35	0.26	0.169
4	Communication Openness	2.03	2.85	2.40	2.58	0.37	1.11	0.088	3.99	2.69	4.16	2.62	0.17	0.57	0.065	0.19	0.20	0.347
5	Patient Counseling	2.72	2.25	3.30	2.19	0.59	1.48	0.043	4.64	2.09	4.66	2.08	0.02	0.39	0.710	0.56	0.24	0.025
6	Staffing, Work Pressure & Pace	−0.90	1.35	−0.77	1.17	0.13	0.72	0.338	0.97	2.12	0.91	2.15	−0.06	0.22	0.095	0.19	0.12	0.119
7	Communication About Prescriptions Across Shifts	0.49	2.12	0.70	1.82	0.21	1.30	0.403	2.48	2.87	2.42	2.90	−0.06	0.91	0.681	0.27	0.27	0.323
8	Communication About Mistakes	1.24	4.22	3.20	3.71	1.95	2.53	<0.001	3.60	4.09	3.44	4.11	−0.16	1.20	0.404	2.11	0.46	<0.001
9	Response to Mistakes	0.40	2.05	1.19	1.99	0.80	1.12	<0.001	2.08	2.64	2.05	2.59	−0.04	0.23	0.324	0.83	0.18	<0.001
10	Organizational Learning—Continuous Improvement	0.15	1.92	1.36	2.20	1.22	1.64	<0.001	2.27	2.88	2.23	2.84	−0.04	0.27	0.324	1.26	0.26	<0.001
11	Overall Perceptions of Patient Safety	1.13	1.55	1.48	1.57	0.35	0.83	0.033	2.37	1.80	2.39	1.78	0.02	0.44	0.772	0.33	0.16	0.039

*S.D.* Standard Deviation, *S.E.* Standard Error

^†^ *p*-values were computed using paired t-test

^‡^ *p*-values were computed using repeated measures analysis of covariance

### Quality-related event reporting

Baseline QRE characteristics were collected during the 30-day pre-intervention period. Analysis indicated that the rate of reported QREs in the intervention and control groups did not change significantly after the intervention. Nonetheless, the pattern of reporting did change in the intervention group. In the intervention group, where QREs were detected and where they occurred changed from pre- to post-intervention (*p* < 0.001). Post-hoc analysis identified that more QREs were identified at the “Partner check” and less in the “DUR” station after the intervention in the intervention group (*p* < 0.001). The analysis also revealed that QREs were reported to occur more in “Assembly of the prescription/filling” (*p* = 0.003) and less in “intervention” (i.e., QREs that require pharmacists to contact prescribers for clarification due to suspected error found in prescriptions) in the intervention group after the intervention (*p* = 0.006). In contrast, the pattern of QRE reporting did not change in the control group.

## Discussion

Guided implementation of the full CQI program improved intervention group respondent attitudes related to patient safety culture in six domains: staff, training, and skill; patient counseling; communication about mistakes; response to mistakes; organizational learning - continuous quality improvement; and overall patient safety perceptions. Communication is a necessary component of reporting QREs and the very first stage of blameless culture. Therefore, perception about communication changed first as it was easier to change in the time frame allotted. It is not surprising when the domain ‘Response to Mistakes’ changed faster because, during training, the researchers emphasized a shameless and blameless approach that focused on systems rather than individuals. The result that domains “Response to Mistakes,” “Organizational Learning – Continuous Improvement,” and “Overall Perception of Patient Safety” changed together is consistent with the fact that they were suspected to share the same latent trait as revealed by factor analysis [21]. The change in percieved safety culture in the intervention group is important because it reflects how well the pharmacy staff in the intervention group adopt the CQI philosophy which is the foundation for implementing a successful CQI program to investigate the causes of medication errors and creating strategic plan to prevent future errors [9, 13].

Although no changes in QRE reporting rates were observed after the intervention, a change in the pattern of QRE reporting was detected after the implementation (i.e., one month after the intervention). While it was hypothesized that after a full CQI implementation intervention groups would demonstrate increased QRE reporting due to better training on how to identify, analyze and prevent mistakes, the rate of QREs reported did not significantly increase in either the control or intervention groups. This result is not entirely unexpected because in another study investigating the impact of a CQI program it took at least one year to see a demonstrable impact of a CQI program in community pharmacies [22]. Therefore, the change in pattern of QRE reporting might indicate that the pharmacy staff analyzed their QREs and made changes to procedures (as was intended by full PQC™ implementation).

In addition, while *more* QRE reporting was expected after full PQC™ implementation perhaps the main impact will be a change in the *mix* of QREs and *how* pharmacy staff members deal with them. While it was usual practice for all pharmacies in this study to record QREs, more than recording is needed to improve quality. That is, intervention group members were trained to not only record QREs, but to then use this information to determine their common problems and to begin to solve some systems issues. Thus, staff members may take action to correct the system and this may, in turn, change the mix of QREs reported as some may be addressed and others may appear. More work is needed to investigate what actions pharmacy staff members take to fix errors, as these were not directly measured in this study.

Several limitations of this study should be noted. First, this study included only a small number of community pharmacies. Therefore, the results may not be generalizable to other settings or to a particular area of the country. Second, it is important to note that although the usual practice in the study pharmacies was to only complete the first 2 steps of the CQI program, it is not necessarily indicative or reflective of the pharmacies in the CQI program. The volunteer pharmacies wanted to improve their performance by fully implementing a CQI program. This could be because they have learned about their QREs trends from the PSO feedback (e.g. newsletters, recommendations) and might have had realized that focused training and getting assistance in more robustly operationalizing their program could be helpful and obtained through participating the study. Third, the followup time (2 months) may not have been long enough to detect the impact of a full CQI implementation on either QREs or patient safety attitudes [23]. Forth, there was the risk of comtamination that control pharmacy staff may accidentally learned about the intervention since the pharmacies were part of a corporate chain and they all were the PSO’s clients. It was possible that contamination occurred through the personal communication from the intervention pharmacists to the control pharmacists or when a pharmacist floated from a treatment to a control pharmacy or vice versa. However, the risk of contamination was minimized by the guided implementation setup including: 1) the guided implementation was provided from the study’s primary investigator; and 2) the training took several hours for intervention pharmacists to be trained in the intervention and only intervention pharmacists received this in-person intensive training. Therefore it was unlikely that the intervention was transmitted through communication between treatment and control pharmacy groups. Even if contamination happened between groups, we were still able to detect a significant improvement in attitude toward safety culture in some domains in the intervention while there was no change in the control group. Thus, the change in the treatment group could have been greater if the contamination was completely prevented.

## Conclusion

The results of this randomized control study suggest that guided implementation of a full CQI program significantly improved self-reported patient safety culture attitudes among pharmacy staff. Additional continuous quality improvement studies in community pharmacies are warranted to assess full impact of safety culture and CQI processes on error rates.

## Additional file

Additional file 1: Rasch item Fit Statistics. (DOCX 121 kb)

## Abbreviations

AHRQ: Agency for Healthcare Research and Quality
ANOVA: Analysis of covariance
APMS: Alliance for Patient Medication Safety
CQI: Continuous quality improvement
DUR: Drug utilization review
IOM: Institute of Medicine
PQC™: Pharmacy Quality Commitment
PSO: Patient Safety Organization
QRE: Quality-related event
SE: Standard error
