# Supplementary material for: Evaluation of a guided continuous quality improvement program in community pharmacies
Source: J Pharm Policy Pract. 2017 Sep 5;10:26. doi: 10.1186/s40545-017-0114-x (PMC5584005; doi:10.1186/s40545-017-0114-x)
Supplement: Additional file 1: — Rasch item Fit Statistics. (DOCX 121 kb) [file 40545_2017_114_MOESM1_ESM.docx]

**Additional file 1.** Rasch item Fit Statistics

|  |  |  | **Pre-intervention**  **(N=69)** | | | |  | **Post-intervention**  **(N=69)** | | | |
| --- | --- | --- | --- | --- | --- | --- | --- | --- | --- | --- | --- |
|  |  | **Domain** | **Item difficulty**  **(logit)** | **S.E.** | **Infit MNSQ** | **Outfit MNSQ** |  | **Item difficulty (logit)** | **S.E.** | **Infit MNSQ** | **Outfit MNSQ** |
| D1 Physical Space and Environment | | |  |  |  |  |  |  |  |  |  |
|  | A1. | This pharmacy is well organized. | -0.85 | 0.23 | 1.04 | 0.91 |  | -1.18 | 0.3 | 1.34 | 1.03 |
|  | A5. | This pharmacy is free of clutter. | 0.47 | 0.17 | 0.69 | 0.81 |  | 0.59 | 0.18 | 0.77 | 0.71 |
|  | A7. | The physical layout of this pharmacy supports good workflow. | 0.38 | 0.38 | 1.35 | 1.01 |  | 0.59 | 0.18 | 1.30 | 1.00 |
| D2 Teamwork | | |  |  |  |  |  |  |  |  |  |
|  | A2. | Staff treat each other with respect. | -0.1 | 0.22 | 0.93 | 0.58 |  | -0.03 | 0.23 | 0.80 | 0.58 |
|  | A4. | Staff in this pharmacy clearly understand their roles and responsibilities. | -0.08 | 0.22 | 1.36 | 1.06 |  | -0.05 | 0.24 | 1.21 | 1.06 |
|  | A9. | Staff work together as an effective team. | 0.18 | 0.21 | 0.67 | 0.56 |  | 0.08 | 0.23 | 0.91 | 0.74 |
| D3. Staff Training and Skills | | |  |  |  |  |  |  |  |  |  |
|  | A3. | Technicians in this pharmacy receive the training they need to do their jobs. | -0.29 | 0.28 | 0.74 | 0.85 |  | -0.39 | 0.29 | 0.61 | 0.66 |
|  | A6. | Staff in this pharmacy have the skills they need to do their jobs well. | -0.49 | 0.28 | **2.39** | **2.87** |  | -0.47 | 0.29 | **2.62** | **2.71** |
|  | A8. | Staff who are new to this pharmacy receive adequate orientation. | 0.89 | 0.22 | 0.77 | 0.75 |  | 0.87 | 0.2 | 0.80 | 0.78 |
|  | A10. | Staff get enough training from this pharmacy. | -0.11 | 0.26 | **0.49** | 0.53 |  | 0 | 0.25 | 0.50 | 0.57 |
| D4. Communication Openness | | |  |  |  |  |  |  |  |  |  |
|  | B1. | Staff ideas and suggestions are valued in this pharmacy. | 0.07 | 0.26 | 1.02 | 0.89 |  | 0.51 | 0.26 | 0.84 | 0.89 |
|  | B5. | Staff feel comfortable asking questions when they are unsure about something. | -0.21 | 0.27 | 1.14 | 0.93 |  | -0.55 | 0.32 | 1.33 | 1.05 |
|  | B10. | It is easy for staff to speak up to their supervisor/ manager about patient safety concerns in this pharmacy. | 0.13 | 0.25 | 0.92 | 0.88 |  | 0.04 | 0.29 | 1.07 | 0.96 |
| D5. Patient Counseling | | |  |  |  |  |  |  |  |  |  |
|  | B2. | We encourage patients to talk to pharmacists about their medications. | -0.03 | 0.3 | 1.29 | 1.10 |  | 0.12 | 0.32 | 1.09 | 1.02 |
|  | B7. | Our pharmacists spend enough time talking to patients about how to use their medications. | 0.85 | 0.27 | 0.74 | 0.71 |  | 0.97 | 0.3 | 0.84 | 0.90 |
|  | B11. | Our pharmacists tell patients important information about their new prescriptions. | -0.82 | 0.33 | 0.84 | 0.83 |  | -1.09 | 0.35 | 0.95 | 0.91 |
| D6. Staffing, Work Pressure & Pace | | |  |  |  |  |  |  |  |  |  |
|  | B3. | Staff take adequate breaks during their shifts. | 0.17 | 0.15 | 1.17 | 1.06 |  | 0.06 | 0.14 | 1.09 | 1.07 |
|  | B9. | We feel rushed when processing prescriptions. (negatively worded) | 0.82 | 0.15 | 0.85 | 0.67 |  | 0.73 | 0.14 | 0.80 | 0.65 |
|  | B12. | We have enough staff to handle the workload. | -2.15 | 0.21 | 0.82 | 0.91 |  | -1.78 | 0.21 | 1.03 | 0.93 |
|  | B16. | Interruptions/distractions in this pharmacy (from phone calls, faxes, customers, etc.) make it difficult for staff to work accurately. (negatively worded) | 1.16 | 0.15 | 0.94 | 0.73 |  | 0.99 | 0.14 | 0.98 | **2.83** |
| D7. Communication About Prescription Across Shifts | | |  |  |  |  |  |  |  |  |  |
|  | B4. | We have clear expectations about exchanging important prescription information across shifts. | -0.19 | 0.26 | 0.87 | 0.75 |  | -0.35 | 0.28 | 0.93 | 0.84 |
|  | B6. | We have standard procedures for communicating prescription information across shifts. | -0.23 | 0.26 | 1.13 | 0.93 |  | -0.22 | 0.26 | 1.25 | 0.99 |
|  | B14. | The status of problematic prescriptions is well communicated across shifts. | 0.42 | 0.22 | 0.89 | 1.14 |  | 0.57 | 0.22 | 0.80 | 0.98 |
| D8. Communication About Mistakes | | |  |  |  |  |  |  |  |  |  |
|  | B8. | Staff in this pharmacy discuss mistakes. | 0.24 | 0.32 | 0.96 | 0.83 |  | -0.09 | 0.35 | 0.99 | 0.71 |
|  | B13. | When patient safety issues occur in this pharmacy, staff discuss them. | -0.52 | 0.32 | 0.92 | 0.70 |  | -0.24 | 0.36 | 0.84 | 0.60 |
|  | B15. | In this pharmacy, we talk about ways to prevent mistakes from happening again. | 0.28 | 0.31 | 1.05 | 0.93 |  | 0.33 | 0.35 | 1.12 | 0.82 |
| D9. Response to Mistakes | | |  |  |  |  |  |  |  |  |  |
|  | C1. | Staff are treated fairly when they make mistakes. | -0.49 | 0.26 | 1.37 | 1.33 |  | -0.45 | 0.25 | 1.43 | 1.32 |
|  | C4. | This pharmacy helps staff learn from their mistakes rather than punishing them. | -0.46 | 0.26 | 0.88 | 0.84 |  | -0.59 | 0.26 | 0.61 | 0.65 |
|  | C7. | We look at staff actions and the way we do things to understand why mistakes happen in this pharmacy. | 0.84 | 0.22 | 0.72 | 0.79 |  | 0.34 | 0.22 | 0.86 | 0.91 |
|  | C8. | Staff feel like their mistakes are held against them. (negatively worded) | 0.12 | 0.24 | 1.15 | 1.18 |  | 0.70 | 0.22 | 1.13 | 0.99 |
| D10. Organizational Learning - Continuous Improvement | | |  |  |  |  |  |  |  |  |  |
|  | C2. | When a mistake happens, we try to figure out what problems in the work process led to the mistake. | -0.13 | 0.2 | 1.27 | 1.11 |  | -0.43 | 0.22 | 1.22 | 1.04 |
|  | C5. | When the same mistake keeps happening, we change the way we do things. | 0.21 | 0.19 | 0.77 | 0.74 |  | 0.39 | 0.20 | 0.68 | 0.69 |
|  | C10. | Mistakes have led to positive changes in this pharmacy. | -0.08 | 0.2 | 0.89 | 0.75 |  | 0.03 | 0.21 | 1.06 | 0.86 |
| D11. Overall Perceptions of Patient Safety | | |  |  |  |  |  |  |  |  |  |
|  | C3. | This pharmacy places more emphasis on sales than on patient safety. (negatively worded) | -0.35 | 0.22 | 1.35 | 1.29 |  | -0.12 | 0.22 | 1.40 | 1.21 |
|  | C6. | This pharmacy is good at preventing mistakes. | 0.66 | 0.18 | 1.02 | 0.94 |  | 0.55 | 0.18 | 1.01 | 0.88 |
|  | C9. | The way we do things in this pharmacy reflects a strong focus on patient safety. | -0.31 | 0.21 | 0.77 | 0.80 |  | -0.42 | 0.23 | 0.77 | 0.81 |

Note: Bold represents Infit or outfit mean square outside reasonable range of 0.5–1.7

MNSQ: Mean square; S.E.: Standard error
